# Hypoxia Imaging Using PET and SPECT: The Effects of Anesthetic and Carrier Gas on [^64^Cu]-ATSM, [^99m^Tc]-HL91 and [^18^F]-FMISO Tumor Hypoxia Accumulation

**DOI:** 10.1371/journal.pone.0025911

**Published:** 2011-11-15

**Authors:** Veerle Kersemans, Bart Cornelissen, Rebekka Hueting, Matthew Tredwell, Kamila Hussien, Philip D. Allen, Nadia Falzone, Sally A. Hill, Jonathan R. Dilworth, Veronique Gouverneur, Ruth J. Muschel, Sean C. Smart

**Affiliations:** 1 Cancer Research United Kingdom/Medical Research Council Gray Institute for Radiation Oncology and Biology, University of Oxford, Oxford, United Kingdom; 2 Chemistry Research Laboratory, University of Oxford, Oxford, United Kingdom; Stanford University, United States of America

## Abstract

**Background:**

Preclinical imaging requires anaesthesia to reduce motion-related artefacts. For direct translational relevance, anaesthesia must not significantly alter experimental outcome. This study reports on the effects of both anaesthetic and carrier gas upon the uptake of [^64^Cu]-CuATSM, [^99m^Tc]-HL91 and [^18^F]-FMISO in a preclinical model of tumor hypoxia.

**Methodology/Principal Findings:**

The effect of carrier gas and anaesthetic was studied in 6 groups of CaNT-bearing CBA mice using [^64^Cu]-CuATSM, [^99m^Tc]-HL91 or [^18^F]-FMISO. Mice were anaesthetised with isoflurane in air, isoflurane in pure oxygen, with ketamine/xylazine or hypnorm/hypnovel whilst breathing air, or in the awake state whilst breathing air or pure oxygen. PET or SPECT imaging was performed after which the mice were killed for organ/tumor tracer quantitation. Tumor hypoxia was confirmed. Arterial blood gas analysis was performed for the different anaesthetic regimes. The results demonstrate marked influences on tumor uptake of both carrier gas and anaesthetic, and show differences between [^99m^Tc]-HL91, [^18^F]-FMISO and [^64^Cu]-CuATSM. [^99m^Tc]-HL91 tumor uptake was only altered significantly by administration of 100% oxygen. The latter was not the case for [^18^F]-FMISO and [^64^Cu]-CuATSM. Tumor-to-muscle ratio (TMR) for both compounds was reduced significantly when either oxygen or anaesthetics (isoflurane in air, ketamine/xylazine or hypnorm/hypnovel) were introduced. For [^18^F]-FMISO no further decrease was measured when both isoflurane and oxygen were administered, [^64^Cu]-CuATSM did show an additional significant decrease in TMR. When using the same anaesthetic regimes, the extent of TMR reduction was less pronounced for [^64^Cu]-CuATSM than for [^18^F]-FMISO (40–60% versus 70% reduction as compared to awake animals breathing air).

**Conclusions/Significance:**

The use of anaesthesia can have profound effects on the experimental outcome. More importantly, all tested anaesthetics reduced tumor-hypoxia uptake. Anaesthesia cannot be avoided in preclinical studies but great care has to be taken in preclinical models of hypoxia as anaesthesia effects cannot be generalised across applications, nor disease states.

## Introduction

Research into the hypoxic tumor microenvironment is accelerating as the importance of tumor hypoxia becomes more and more apparent. Most solid tumors develop regions of hypoxia as they grow and evidence from experimental and clinical studies points to a significant role for tumor hypoxia in tumor propagation, resistance to radio- and chemotherapy and malignant progression [1]. As the presence of tumor hypoxia represents a barrier for effective cancer treatment, identifying patients whose tumors contain hypoxic areas will therefore have an important role in tumor prognosis, treatment and outcome.

The current gold standard to measure tissue oxygen concentration, and thus tumor hypoxia, is the use of oxygen-sensitive electrodes which determine the oxygen partial pressure pO_2_ [2]. However, sampling errors are easily introduced and given the invasive nature of the method, it is difficult to reach deep-seated tumors. An elegant alternative could be presented by using nuclear imaging techniques.

Over the years, imaging as a noninvasive method has attracted a lot of attention and several radiotracers have been developed for the evaluation of hypoxia using Positron Emission Tomography (PET) and Single Photon Emission Computed Tomography (SPECT). Examples of such compounds include the nitroimidazoles such as [^18^F]fluoromisonidazole ([^18^F]-FMISO) and [^123^I]iodoazomycin arabinoside (IAZA) and non-nitroimidazole compounds such as [^64^Cu]diacetyl-bis(*N*4-methylthiosemicarbazone) ([^64^Cu]-CuATSM) and [^99m^Tc]butylene amineoxime ([^99m^Tc]-HL91) [3]. To date, more and more candidates are being developed to improve hypoxia targeting characteristics. However, a direct comparison of compounds is often difficult mainly due to a wide variety in experimental set ups, an important component of which is the anaesthetic protocol.

While anaesthesia is preferred in preclinical imaging studies to reduce subject movement, anaesthetic agents, and their carrier gasses in case of volatile anaesthetics, can influence radiotracer biodistribution and tumor uptake substantially. This is well known for neurological applications and many reports describe the effect of anaesthetics, mostly barbiturates, on brain function [4], [5], [6]. However, less is published on the general impact of anaesthesia on extracerebral metabolism, especially in nuclear medicine applications. Zanelli et al described the influence of pentobarbital on blood perfusion in transplanted mouse tumors. Using ^86^Rb, ^125^I-human serum albumin and ^51^Cr-labeled red blood cells, pentobarbital was found to both increase the relative blood perfusion in tumors and kidneys and to decrease the relative muscle perfusion, but to reduce the blood volume of the tumor and kidneys [7]. It was only in 2005 that Lee et al. studied the effect of ketamine/xylazine and pentobarbital on the biodistribution and tumor uptake of the most abundantly used tracer in nuclear medicine: [^18^F]-Fluorodeoxyglucose ([^18^F]-FDG) [8]. Both ketamine/xylazine and pentobarbital sedation increased blood [^18^F]-FDG activity resulting in decreased tumor to muscle ratios and PET image contrast. A few years later, these findings were extended for isoflurane and sevoflurane in both air and oxygen by Flores and co-workers who demonstrated that both the anaesthetic drug and the carrier gas can influence [^18^F]-FDG tumor uptake [9].

As both organ perfusion and function are often changed by anaesthesia, it seems obvious to test its effects on tumor hypoxia as well. Although the effects of anaesthetics on *in vivo* oxygen electrode measurements have been examined, so far no papers have considered its effects on radiodiagnostic markers [10]. Moreover, no consensus for the optimal anaesthetic protocol exists for preclinical hypoxia imaging using agents such as [^99m^Tc]-HL91, [^64^Cu]-CuATSM or [^18^F]-FMISO, albeit an Investigational New Drug Applications (IND) was filed for the latter one. Although the anaesthesia method is kept constant throughout each study, different studies use different protocols which differ in the anaesthetic drugs used (isoflurane versus pentobarbital and ketamine/xylazine), and in the carrier gases (oxygen, air or a mixture) and the administration routes (inhalation, i.p. or i.m.). The huge amount of existing data on hypoxia imaging, together with the filed IND for [^18^F]-FMISO point out the importance of standardizing preclinical hypoxia imaging regimes across studies.

This report describes for the first time the impact of the anaesthetic protocol on [^18^F]-FMISO, [^64^Cu]-CuATSM and [^99m^Tc]-HL91 uptake in hypoxic tumors for imaging applications using PET or SPECT.

## Materials and Methods

### Ethics Statement

All animal studies were performed in accordance with the Animals Scientific Procedures Act of 1986 (UK) (Project License Number 30/2514 issued by the Home Office).

#### Radiopharmaceuticals

[^18^F]-FMISO (specific activity = 115 GBq/µmol) was obtained from the Wolfson Brain Imaging Centre, Addenbrookes Hospital, Cambridge.

[^99m^Tc]-HL91 was prepared as follows. HL91 solution (28 µL, 2 mg/mL), tartrate solution (20 µL, 10 mg/mL; Sigma-Aldrich) and sodium carbonate buffer solution (100 µL, pH 10.0, 0.1 mol/L; Sigma-Aldrich) were mixed, immediately followed by the addition of [^99m^TcO_4_]^−^ (100–150 MBq) and freshly prepared SnCl_2_ solution (25 µL, 1 mg/mL in 1 M HCl; Sigma-Aldrich). This mixture was incubated for 30 min at room temperature. Thereafter, 50% acetonitrile aqueous solution was used to determine [^99m^Tc]-colloid on Whatman No.1 paper strip (Rf = 0; Rf [^99m^Tc]-HL91 = 1) and water to determine pertechnetate (Rf = 1.0; Rf [^99m^Tc]-HL91 = 0). The radiochemical yield for [^99m^Tc]-HL91 was 96%±7% and no further purification was needed which resulted in a specific activity of 3.5–5 MBq/µg.

Copper-64 was purchased from the Wolfson Brain Imaging Centre, Addenbrookes Hospital, Cambridge or from the PET Imaging Centre, St Thomas' Hospital, London, UK. [^64^Cu]-CuATSM was prepared from H_2_ATSM as previously described [11]. Briefly, to 40 µL of H_2_ATSM stock solution (1 mg/mL) was added 50 µL of dimethyl sulfoxide (DMSO) and 50 µL of aqueous [^64^Cu]Cu(OAc)_2_ (100 MBq). This was loaded onto a C-18 Sep-Pak Light®, pre-equilibrated with 2 mL ethanol (EtOH) and 10 mL H_2_O. After loading the sample, 5 mL H_2_O was passed through to remove the DMSO and any unreacted Copper-64. The labelled complex was eluted in EtOH (600 µL, following a 200 µL void volume). The EtOH was concentrated under a stream of nitrogen and diluted with 0.9% saline solution to give a <10% EtOH solution for injection. [^64^Cu]-CuATSM was prepared in 90% isolated radiochemical yield, the radiochemical purity was >98% as determined by radio-TLC on silica gel plates using ethyl acetate/methanol (95∶5) as the mobile phase. The specific activity of the administered radiopharmaceutical was 2–3 MBq/µg of labelling precursor.

#### Animal model

Water and food were freely available during the experimental period. The murine adenocarcinoma NT (CaNT) was implanted subcutaneously onto the right thigh of 6–7 week-old female CBA mice. Fifty µL of a crude cell suspension, prepared by mechanical dissociation of an excised tumor from a donor animal, was injected. Tumors were selected for imaging when the geometric mean diameter reached 6–8 mm, approximately 3 weeks after implantation. Throughout the experiments, mice were maintained at 37°C and respiration rate was monitored (60–90 respirations/minute for imaging and 30–60 respirations/minute for blood gas analysis).

#### Anaesthesia and experimental set-up

For [^18^F]-FMISO, [^64^Cu]-CuATSM and [^99m^Tc]-HL91, the effects of anaesthesia on tumor uptake and biodistribution were studied in 6 groups (4 groups for [^64^Cu]-CuATSM) of CaNT bearing CBA mice (n = 9 per group). Anaesthesia was induced and maintained using either gases (isoflurane in oxygen (IO) or in room-air (IA)), or injectable agents (hypnorm/hypnovel (HHA) or Ketamine/Xylazine (KXA), both breathing room air). Non-anaesthetised mice were kept in room air (UA) or in a 100% oxygen atmosphere (UO). An overview of the protocols used is presented in Table 1. PET or planar SPECT imaging was performed for 2 h on all anaesthetised mice following injection of the radiolabeled compound.

**Table 1:** Schematic representation of the anaesthesia regimes used.

Group	Anaesthesia induction	Anaesthesia maintenance
IO*	3% isoflurane in 100% oxygen	1.5% isoflurane in 100% oxygen
IA†	3% isoflurane in room air	1.5% isoflurane in room air
HHA‡	Hypnorm/H_2_O/Hypnovel1∶2∶1 at 10 ml/kg (i.p.)	Hypnorm/H_2_O/Hypnovel1∶2∶1 at 0.3 ml/kg (i.p.)
KXA§	Ketamine/Xylazine80 mg/kg : 10 mg/kg (i.p.)	Ketamine/Xylazine8 mg/kg : 1 mg/kg (i.p.)
UO∥	Not applicable: no imaging performed, only dissection.100% oxygen atmosphere during radiotracer distribution	
UA¶	Not applicable: no imaging performed, only dissection.room air atmosphere during radiotracer distribution	

*Anaesthetised using Isoflurane in Oxygen;

† Anaesthetised using Isoflurane in room air;

‡ Anaesthetised using Hypnorm/Hypnovel;

§ Anaesthetised using Ketamine/Xylazine;

∥ Awake breathing oxygen;

¶ Awake breathing room air.

At 2 hours p.i., all mice from all anaesthetic regimes (IO, IA, HHA, KXA, UO and UA) were sacrificed by cervical dislocation. The organs and tissues were removed, washed and weighed. The blood was collected and weighed. The tumor was collected, weighed and flash frozen. The radioactivity of all samples was counted by use of an automated gamma-counting system (Perkin Elmer). The amount of radioactivity in the organs and tissues was decay corrected and calculated as % injected dose per gram (%ID/g): [(activity_tissue_)/(weight_tissue_×activity_injected_)].

### Small animal imaging

#### PET/CT

PET imaging was performed using the Inveon PET/CT system (Siemens Preclinical Solutions) equipped with a custom built imaging cradle. Computed Tomography (CT) based attenuation correction was performed before each PET emission scan and was also used for anatomical referencing. Two hour whole-body dynamic scans were acquired. Anaesthesia was induced, mice were placed supine, head first, in the imaging cradle and a cannula (30-gauge needle attached to PE-10 tubing) was inserted into the lateral tail vein. Following the attenuation CT-scan, 10 MBq of [^18^F]-FMISO or [^64^Cu]-CuATSM was injected through the cannula immediately after the PET emission data acquisition was initiated. Data were histogrammed in 21 time frames (6× 15s, 3× 60s, 5× 300s, 3×600s and 4× 900s) and global deadtime correction and Fourier rebinning were applied. The dynamic histograms were reconstructed using a 2-dimensional filtered backprojection algorithm, a Ramp projection filter, a 0.5/mm Nyquist projection cut-off value, no zoom and a matrix size of 128×128×159 (sagittal×coronal×transversal). ROI image analysis was performed using the Inveon Research Workplace software (IRW, version 2.2, Siemens Preclinical Solutions).

#### SPECT

Planar SPECT imaging was performed using the nanoSPECT/CT system (Bioscan) equipped with an Ultra High Resolution (UHR) parallel hole collimator. Anaesthesia was induced, 4 mice were placed simultaneously, supine, head first, on the collimator and a cannula was inserted into their lateral tail vein. Ten MBq of [^99m^Tc]-HL91 was injected through the cannula immediately after the two hour whole-body dynamic scans were initiated. Fifty-five time frames (30× 20s, 10× 60s, 10× 300s and 5× 600s) were acquired. ROI image analysis was performed using ImageJ [12].

#### Blood gas analysis

Non-tumor bearing CBA mice (n = 5 per anaesthetic regime) were anaesthetised using the different anaesthesia regimes (IO, IA, HHA and KXA). Femoral cannulation (PE10 tubing) was performed to allow for arterial blood gas analysis. A blood sample (50 µl) was collected at 90 min post anaesthesia induction and pO_2_, pCO_2_ and sO_2_ was determined using an I-Stat blood gas analyser (Model 300, Abbott).

#### Tumor hypoxia

The hypoxic status of all tumors was confirmed using the OxyLite probe (Oxford Optronix Ltd), which provided continuous real-time tissue pO_2_ measurements at the centre of the tumor. Immunohistological staining for EF5 (2-(2-nitro-1H-imidazol-1-yl)-*N*-(2,2,3,3,3-pentafluoropropyl)acetamide; University of Pennsylvania) as described by Lee J et al was performed on a subset of tumors [13]. For EF5 studies, tumor bearing mice from each anaesthetic group (n = 3) were administered 200 µl of 10 mM EF5 in 0.9% saline i.v. 3 h prior to tumor excision (EF5 was obtained from Dr. Cameron Koch, University of Pennsylvania, Philadelphia, PA). Sections were stored in 1% paraformaldehyde at 4°C and images were acquired within 2 days of staining. Fluorescence detection was performed at ×10 magnification with an upright motorised Nikon Eclipse 90i system (Nikon, UK), fitted with a motorised stage and equipped with cooled charge-coupled Hamamatsu ORCA-ER camera and acquisition software.

#### Statistical analysis

Tumor uptake of [^18^F]-FMISO, [^64^Cu]-CuATSM or [^99m^Tc]-HL91 in vivo was compared for different anaesthesia groups by 1-way parametric ANOVA using the Tukey adjustment for multiple comparisons (*P*<0.05). Correlations between biodistribution and imaging results were made using a Pearson correlation test (*P*<0.05). All other statistical comparisons were made using a Student *t* test (*P*<0.05).

## Results

**Figure 1:**
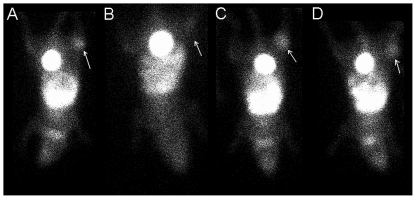
Representative planar SPECT images of the uptake of [^99m^Tc]-HL91 in CaNT bearing CBA mice at steady state (2 h p.i.). The tumour is indicated by the white arrow. (A) IA: Anaesthetised using Isoflurane in room air; (B) IO: Anaesthetised using Isoflurane in Oxygen; (C) HHA: Anaesthetised using Hypnorm/Hypnovel; (D) KXA: Anaesthetised using Ketamine/Xylazine.

**Figure 2:**
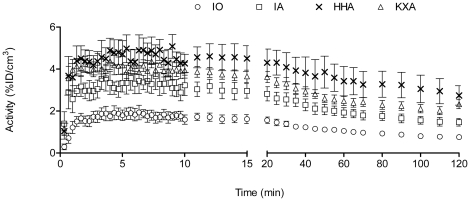
Overall mean tumor uptake for [^99m^Tc]-HL91 as a function of time by dynamic planar SPECT imaging. HHA: Anaesthetised using Hypnorm/Hypnovel; IA: Anaesthetised using Isoflurane in room air; IO: Anaesthetised using Isoflurane in Oxygen; KXA: Anaesthetised using Ketamine/Xylazine.

**Figure 3:**
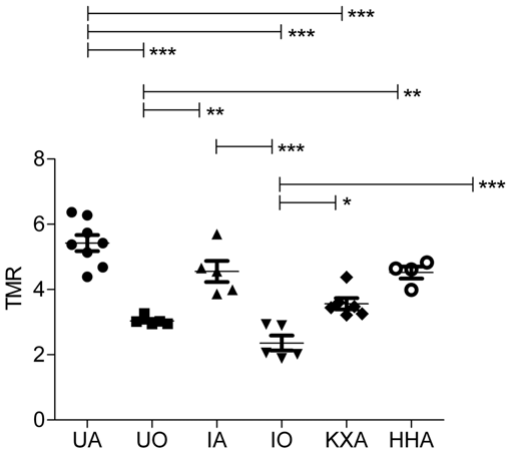
Tumor-to-muscle ratio for [^99m^Tc]-HL91 for all tested anaesthetic regimes. HHA: Anaesthetised using Hypnorm/Hypnovel; IA: Anaesthetised using Isoflurane in room air; IO: Anaesthetised using Isoflurane in Oxygen; KXA: Anaesthetised using Ketamine/Xylazine; TMR: tumor-to-muscle ratio; UA: Awake breathing room air; UO: Awake breathing oxygen.

Planar SPECT imaging using a UHR collimator allowed for very fast 2D imaging, resulting in images with acceptable resolution (2.0 mm, tested in-house). Although one spatial dimension was lost and CT was not possible, temporal resolution was gained and thus kinetic analysis of [^99m^Tc]-HL91, the most studied radiotracer for hypoxia imaging using SPECT, was performed. Four mice were imaged simultaneously using the anaesthesia protocols as described in Table 1 and a representative planar SPECT image for each condition at 2 h p.i. is shown in Figure 1. The resulting biodistribution kinetics for tumor and the tumor-to-muscle ratios (TMR) are presented in Figure 2 and 3, respectively. More detailed dissection analysis was performed when steady state for [^99m^Tc]-HL91 tumor uptake was reached at 2 h p.i. and these results, expressed as %ID/g for each organ, are shown in Table 2. Comparable TMR were obtained for awake animals breathing air and for those anaesthetised with isoflurane in air or hypnorm/hypnovel. However, when additional oxygen was introduced to the system, either for awake or anaesthetised mice, the TMR value was reduced significantly (p<0.001). The same was true when a ketamine/xylazine mixture was used. No substantial changes in TMR were detected when only isoflurane, and not oxygen, was supplied. The effects of anaesthesia on the tumor-to-blood ratio (TBR) showed a different pattern, namely: IO = UO<UA = IA<KXA = HHA. In other words, the TBRs for both injectable anaesthetics were highest whereas those for oxygen as a carrier gas were lowest and the latter regardless of the use of isoflurane.

**Table 2:** Biodistribution results for [^99m^Tc]-HL91 at 2 h p.i. in a CaNT bearing CBA mouse model.

	IO*	IA†	HHA‡	KXA§	UA∥	UO¶
Blood	2.4±0.1	5.7±0.9	3.7±0.5	3.6±0.3	2.4±0.6	2.7±0.2
Tumor	1.3±0.1	2.6±0.5	3.9±0.7	4.3±0.1	2.2±0.5	1.2±0.0
Muscle	0.5±0.1	0.7±0.1	0.9±0.3	0.9±0.1	0.4±0.1	0.4±0.0
Skin	1.1±0.4	1.1±0.3	1.5±0.1	1.8±0.4	0.8±0.4	0.9±0.2
Stomach	1.5±0.4	3.4±0.8	19.6±12.4	14.4±4.5	4.1±2.4	3.2±0.4
Small intestine	17.6±3.1	10.2±3.6	21.8±14.9	7.7±1.1	2.7±1.1	3.2±0.4
Large intestine	3.9±1.9	4.8±0.7	11.0±0.9	5.1±1.3	14.4±3.2	12.0±1.8
Fat	0.9±0.6	1.8±0.5	2.5±0.2	2.7±0.5	0.7±0.1	0.6±0.1
Spleen	2.2±0.7	2.0±0.4	7.6±0.8	10.0±2.1	6.2±1.5	4.9±0.6
Liver	9.9±1.6	9.5±1.6	9.6±0.8	7.8±1.3	10.7±3.4	12.7±1.7
Kidneys	7.8±4.9	9.1±2.4	9.8±4.1	7.7±2.1	5.1±1.1	6.7±0.8
Heart	1.1±0.1	2.1±0.4	1.99±0.3	2.0±0.7	0.9±0.1	1.1±0.1
Lungs	1.7±0.2	4.5±0.7	4.1±0.7	2.6±0.5	1.9±0.4	1.5±0.1
TMR	2.4±0.6	4.6±0.7	4.7±0.5	3.6±0.5	4.8±0.8	3.0±0.1
TBR	0.5±0.1	0.8±0.3	1.2±0.2	1.3±0.3	0.9±0.2	0.4±0.1

Results are expressed as average % injected dose per gram tissue ± standard deviation. TMR = ratio tumor-to-muscle, TBR = ratio tumor-to-blood.

* Anaesthetised using Isoflurane in Oxygen;

† Anaesthetised using Isoflurane in room air;

‡ Anaesthetised using Hypnorm/Hypnovel;

§ Anaesthetised using Ketamine/Xylazine;

∥ Awake breathing room air;

¶ Awake breathing oxygen.

**Figure 4:**
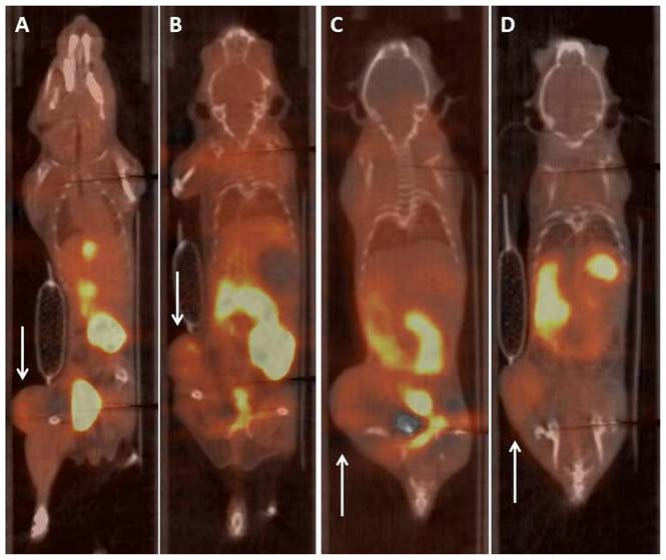
Representative PET/CT images of the uptake of [^18^F]-FMISO in CaNT bearing CBA mice at steady state (2 h p.i.). (A) IA: Anaesthetised using Isoflurane in room air; (B) IO: Anaesthetised using Isoflurane in Oxygen; (C) HHA: Anaesthetised using Hypnorm/Hypnovel; (D) KXA: Anaesthetised using Ketamine/Xylazine. T = tumour, B = bladder, L = Liver.

**Figure 5:**
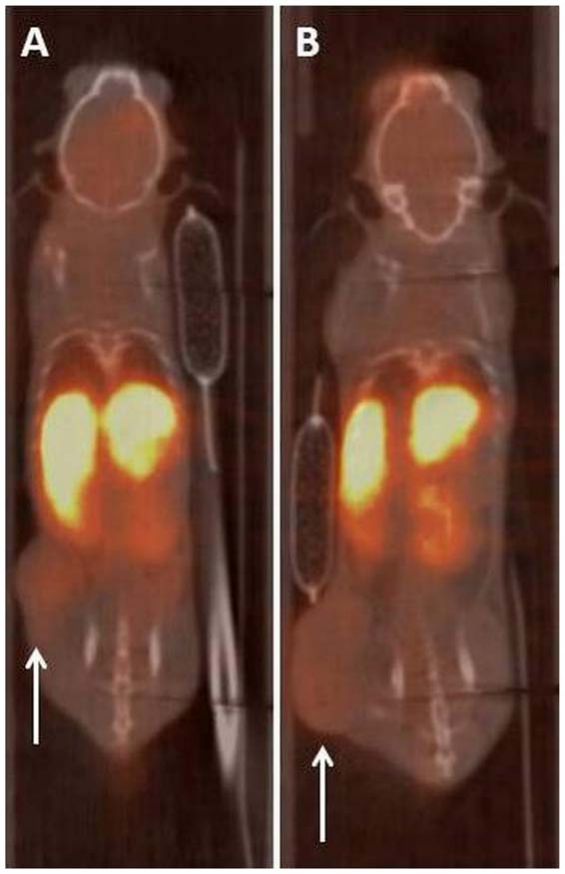
Representative PET/CT images of the uptake of [^64^Cu]-CuATSM in CaNT bearing CBA mice at steady state (2 h p.i.). (A) IA: Anaesthetised using Isoflurane in room air; (B) IO: Anaesthetised using Isoflurane in Oxygen.

**Figure 6:**
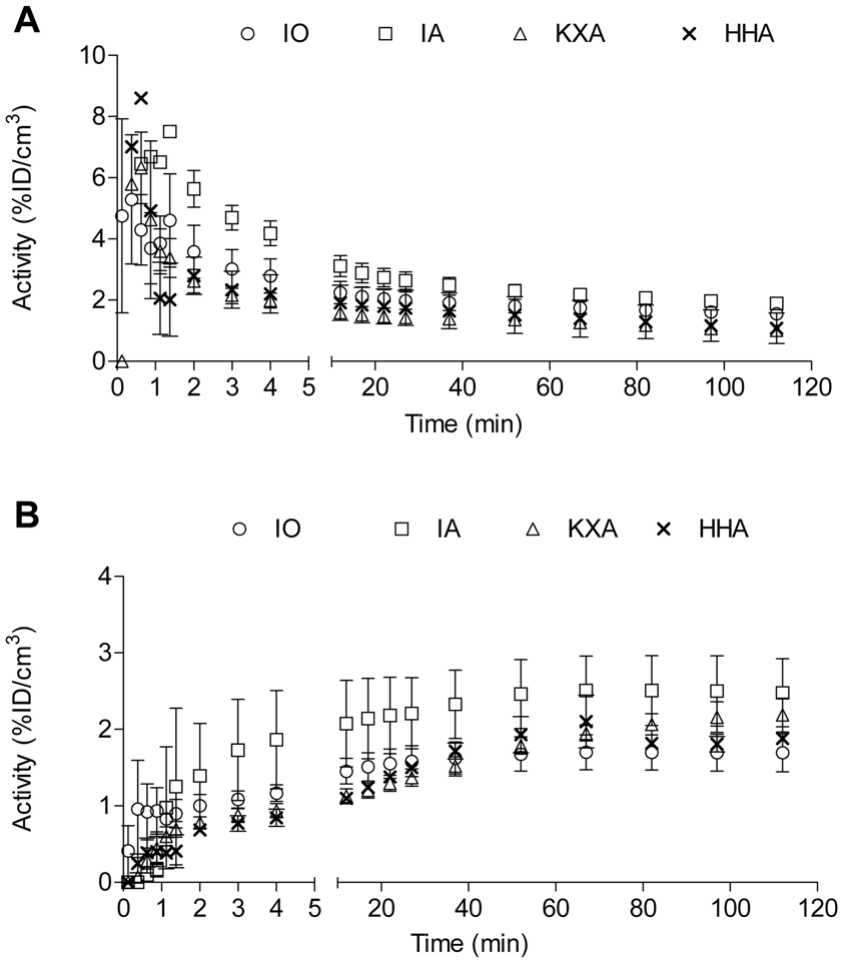
Overall mean heart and tumor uptake for [^18^F]-FMISO as a function of time by kinetic PET imaging. (A) Heart and (B) Tumour uptake. HHA: Anaesthetised using Hypnorm/Hypnovel; IA: Anaesthetised using Isoflurane in room air; IO: Anaesthetised using Isoflurane in Oxygen; KXA: Anaesthetised using Ketamine/Xylazine.

**Figure 7:**
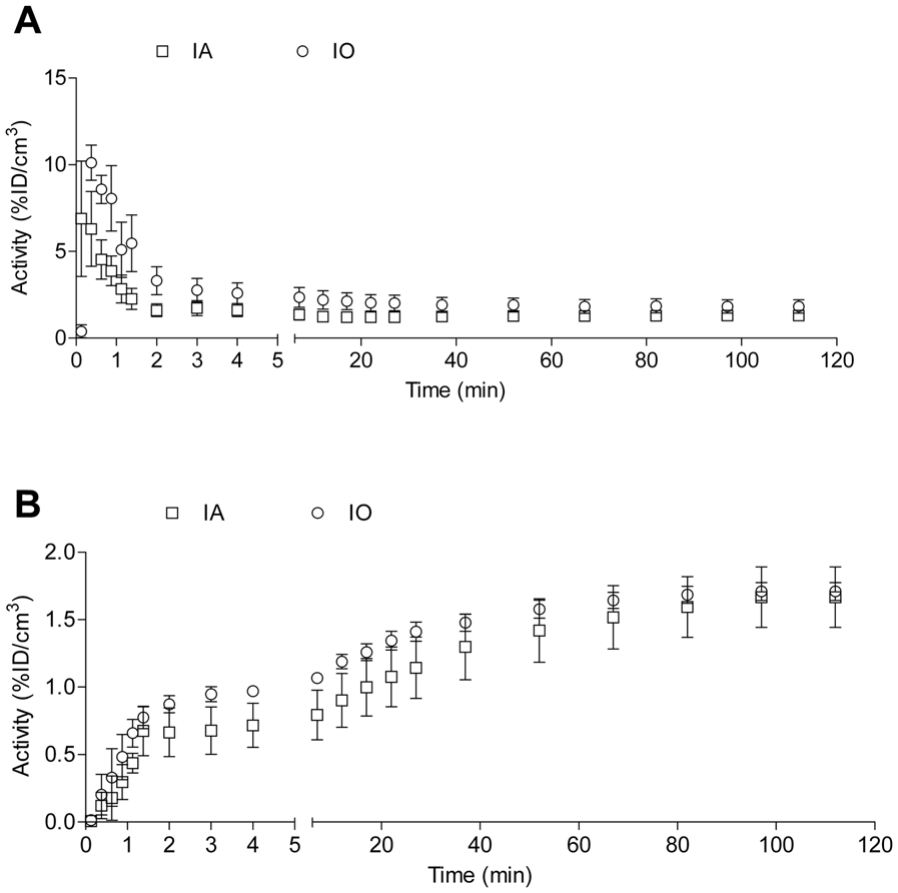
Overall mean heart and tumor uptake for [^64^Cu]-CuATSM as a function of time by kinetic PET imaging. (A) Heart and (B) Tumour uptake. IA: Anaesthetised using Isoflurane in room air; IO: Anaesthetised using Isoflurane in Oxygen.

**Figure 8:**
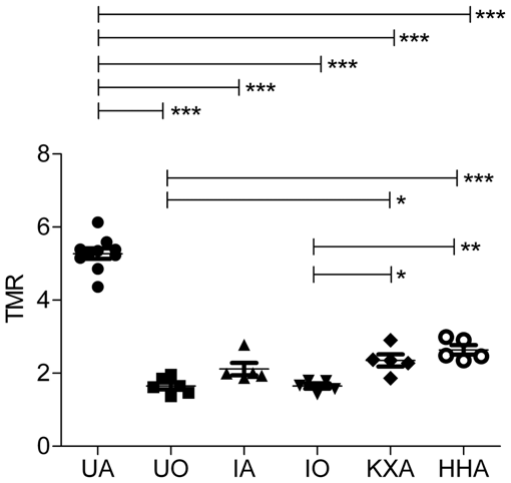
Tumor-to-muscle ratio for [^18^F]-FMISO for all tested anaesthetic regimes. HHA: Anaesthetised using Hypnorm/Hypnovel; IA: Anaesthetised using Isoflurane in room air; IO: Anaesthetised using Isoflurane in Oxygen; KXA: Anaesthetised using Ketamine/Xylazine; TMR: tumor-to-muscle ratio; UA: Awake breathing room air; UO: Awake breathing oxygen.

**Figure 9:**
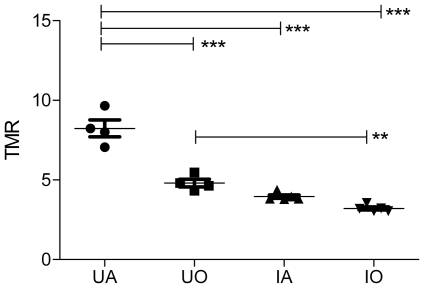
Tumor-to-muscle ratio for [^64^Cu]-CuATSM for all tested anaesthetic regimes. IA: Anaesthetised using Isoflurane in room air; IO: Anaesthetised using Isoflurane in Oxygen; TMR: tumor-to-muscle ratio; UA: Awake breathing room air; UO: Awake breathing oxygen.

Nevertheless, most hypoxia imaging agents are not being developed for SPECT but for PET [3]. The latter technique benefits from list-mode data acquisition resulting in high temporal resolution with full 3D spatial resolution. To date, [^18^F]-FMISO and [^64^Cu]-CuATSM are the lead contenders for in vivo assessment of hypoxia in humans [14], [15]. As these compounds belong to two distinct classes: the nitroimidazoles and the bis(thiosemicarbazonates), and exploit different tumor localisation mechanisms, it was important to include both in this study [16]. Representative PET images for [^18^F]-FMISO and [^64^Cu]-CuATSM for each condition are depicted in Figures 4 and 5, respectively. The blood input function and dynamic tumor uptake of [^18^F]-FMISO and [^64^Cu]-CuATSM as a function of time is illustrated in Figures 6A, 6B, 7A and 7B. The resulting TMRs are presented in Figure 8 and 9, including those for the awake state. Dissection results for each organ at 2 h p.i. are shown in Tables 3 and 4. The TMR for both compounds was reduced significantly when only oxygen or any anaesthetic were used as compared to awake mice breathing air (p<0.001). However, more subtle differences exist for [^64^Cu]-CuATSM. Indeed, the effects of isoflurane or oxygen alone could not be distinguished from each other when using [^18^F]-FMISO as a tumor hypoxia imaging agent. Yet for [^64^Cu]-CuATSM, the TMR was reduced when oxygen was added to the breathing mixture, but even more so when isoflurane was supplemented. Again, the results for TBR diverge slightly from those for TMR for both [^18^F]-FMISO and [^64^Cu]-CuATSM, and show a different pattern as compared to [^99m^Tc]-HL91. Briefly, TBR could be ranked as follows: IO = IA<UO = KXA = HHA<UA.

**Table 3:** Biodistribution results for [^18^F]-FMISO at 2 h p.i. in a CaNT bearing CBA mouse model.

	IO*	IA†	HHA‡	KXA§	UA∥	UO¶
Blood	2.7±0.4	2.3±0.2	1.7±0.2	1.8±0.2	1.2±0.1	1.3±0.2
Tumor	4.5±0.4	3.9±0.4	7.4±0.9	5.7±0.7	6.5±0.7	4.1±0.5
Muscle	2.7±0.3	1.9±0.3	1.4±0.1	1.6±0.1	1.2±0.1	2.5±0.2
Skin	0.6±0.1	0.6±0.0	0.7±0.1	0.6±0.1	0.6±0.0	0.6±0.1
Stomach	1.6±0.7	2.8±0.4	2.2±0.7	2.7±0.4	2.2±0.3	1.4±0.2
Small intestine	6.5±0.3	3.9±0.4	5.4±1.1	7.0±0.6	5.9±1.7	2.7±0.2
Large intestine	14.1±1.8	9.4±0.9	19.0±2.3	14.5±3.1	15.3±2.7	21.0±2.2
Fat	0.7±0.0	0.7±0.1	0.6±0.1	0.6±0.1	0.8±0.1	1.4±0.3
Spleen	2.3±0.2	2.4±0.2	1.5±0.3	1.9±0.1	1.4±0.1	1.9±0.2
Liver	3.9±0.5	2.7±0.3	3.5±0.4	9.2±1.0	3.8±0.7	2.6±0.2
Kidneys	4.0±0.5	3.7±0.3	2.9±0.1	3.6±0.4	2.5±0.3	2.1±0.2
Heart	3.7±0.4	3.6±0.4	2.5±0.2	2.2±0.2	1.6±0.2	2.9±0.3
Lungs	2.5±0.4	3.0±0.6	2.2±0.2	2.0±0.2	1.9±0.2	2.1±0.2
TMR	1.7±0.2	2.2±0.4	5.2±0.7	3.5±0.7	5.3±0.6	1.7±0.2
TBR	1.7±0.2	1.8±0.3	4.0±0.9	3.3±0.2	5.6±0.6	3.0±0.4

Results are expressed as average % injected dose per gram tissue ± standard deviation. TMR = ratio tumor-to-muscle, TBR = ratio tumor-to-blood.

*Anaesthetised using Isoflurane in Oxygen;

† Anaesthetised using Isoflurane in room air;

‡ Anaesthetised using Hypnorm/Hypnovel;

§ Anaesthetised using Ketamine/Xylazine;

∥ Awake breathing room air;

¶ Awake breathing oxygen.

**Table 4:** Biodistribution results for [^64^Cu]-CuATSM at 2 h p.i. in a CaNT bearing CBA mouse model.

	IO*	IA†	UA‡	UO§
Blood	0.9±0.1	0.8±0.1	1.2±0.0	1.2±0.1
Tumor	1.1±0.1	1.4±0.0	3.1±0.3	2.3±0.0
Muscle	0.3±0.0	0.3±0.0	0.4±0.0	0.5±0.1
Skin	0.4±0.0	0.5±0.0	0.4±0.0	0.4±0.0
Stomach	6.1±1.0	4.7±0.5	3.9±0.6	6.0±0.7
Small intestine	9.9±0.5	9.7±0.3	4.7±0.6	9.9±0.2
Large intestine	3.3±0.3	3.7±0.3	11.9±1.1	5.6±0.1
Fat	0.5±0.0	0.5±0.1	0.4±0.1	0.5±0.1
Spleen	1.7±0.1	1.7±0.1	1.5±0.2	1.6±0.1
Liver	11.7±0.8	10.6±0.3	6.8±0.3	6.9±0.8
Kidneys	5.9±0.5	6.2±0.8	4.8±0.2	5.0±0.5
Heart	1.5±0.2	1.3±0.1	2.1±0.1	2.3±0.2
Lungs	5.2±0.3	5.8±0.4	5.8±0.5	4.6±0.3
TMR	3.6±0.2	4.0±0.3	8.2±1.3	4.8±0.6
TBR	1.2±0.2	1.8±0.1	2.6±0.4	1.9±0.1

Results are expressed as average % injected dose per gram tissue ± standard deviation. TMR = ratio tumor-to-muscle, TBR = ratio tumor-to-blood.

*Anaesthetised using Isoflurane in Oxygen;

† Anaesthetised using Isoflurane in room air;

‡ Awake breathing room air;

§ Awake breathing oxygen.

Although the CaNT bearing CBA mouse model is very robust in terms of tumor growth and tumor hypoxia status throughout tumor development, the presence and level of hypoxia was confirmed before mice entered the study using OxyLite measurements (average pO_2_ for mouse population: 2.3 mmHg±0.9 mmHg) . Following imaging and prior to culling the mice for dissection analysis, additional pO_2_ readings were performed. The latter resulted in average tumor pO_2_ values for IO, IA, HHA, KXA, UA and UO of 1.5 mmHg±0.4 mmHg, 1.9 mmHg±0.6 mmHg, 1.4 mmHg±0.5 mmHg, 2.4 mmHg±0.4 mmHg, 0.9 mmHg±0.3 mmHg and 1.1 mmHg±0.3 mmHg. No significant differences were observed for the different anaesthesia regimes. Additionally, immunohistological EF5 staining on tumors from each anaesthetic group showed no differences in the extent of hypoxia for IA, HHA, KXA and UA. However, when oxygen was used as a carrier gas (IO and UO) the latter was largely reduced.

As variations in arterial oxygen concentrations can lead to acute hypoxia, it is important to check the arterial pO_2_, pCO_2_ and blood oxygen saturation levels (sO_2_). Arterial blood sampling was performed and the results summarised in Table 5. No significant differences could be detected for sO_2_ and pCO_2_ for the different anaesthesia regimes. However, pO_2_ was significantly higher only when isoflurane in 100% oxygen was used. Measurements could not be performed for the UO and UA groups due to the necessary surgical intervention.

**Table 5:** Arterial pO_2_, pCO_2_ and sO_2_ levels at 90 min post anaesthesia induction for different anaesthesia regimes (n = 5/group).

Group	pO_2_ (mmHg)	pCO_2_ (mmHg)	sO_2_ (%)
IO*	434±68	60.2±0.4	100±0
IA†	103±13	58.2±6.0	98±1
HHA‡	95±7	60.6±8.1	94±4
KXA§	94±4	60.6±1.5	95±3

Results are expressed as average ± standard deviation.

*Anaesthetised using Isoflurane in Oxygen;

† Anaesthetised using Isoflurane in room air;

‡ Anaesthetised using Hypnorm/Hypnovel;

§ Anaesthetised using Ketamine/Xylazine.

## Discussion

Tissue hypoxia is studied in many medical disciplines as it is a prognostic marker associated with an aggressive clinical and biological phenotype [17]. However, despite its occurrence in other pathologic conditions, including myocardial ischemia and stroke, hypoxia imaging is much more advanced in oncology applications where it is used to predict response to therapy and to assess overall prognosis. In order to provide *a priori* information on tumor hypoxia, the oxygenation status of solid tumors has been evaluated extensively using a wide variety of both invasive and non-invasive techniques [18], [19]. However, non-invasive techniques that permit both serial imaging and the detection of tumor hypoxia levels could provide valuable information on disease status and on treatment response. As a result, the search for a non-invasive hypoxia assay has been continuous and various PET and SPECT compounds have been developed and are being evaluated in vivo in small animal models [3]. While anaesthesia is preferred in preclinical imaging studies to reduce motion artefacts, anaesthetic agents, and their carrier gases, in the case of volatile anaesthetics, have the potential to alter the study outcome dramatically. Therefore, it is important to study the effects of anaesthesia and come to a consensus imaging protocol before embarking on a programme of in vivo experiments.

In this study, the CaNT bearing CBA mouse model was used and 2 different methods were applied to confirm the tumor hypoxic status, i.e. immunohistological EF5 staining and pO_2_ OxyLite measurement. Both techniques showed the presence of tumor hypoxia throughout tumor development (data not shown). Although the pO_2_ measurements were limited in sample size, it was a simple and fast method to get a good estimate of the hypoxia status of the tumors, i.e. is hypoxia present or not. As well as considering the effects of the anaesthetic, the imaging set-up needs validation to avoid introducing other biases. Changes in core temperature have the potential to alter various physiological factors including blood perfusion, pO_2_, vascular permeability and intratumoral pressure. Furthermore, mild hyperthermia improves tumor oxygenation in rodents [20]. For these reasons, all animals were placed on a heating pad and a negative feedback loop connected to a rectal probe used to ensure a stable core temperature of 37°C.

Having validated our experimental set-up, the effects of anaesthesia could be studied in detail. The results presented in this study not only established the marked influences on tumor uptake of both carrier gas and anaesthetic, but also demonstrated pronounced differences between [^99m^Tc]-HL91, [^18^F]-FMISO and [^64^Cu]-CuATSM despite the fact they are all designed to target hypoxia specifically. Thus the effects of isoflurane and hypnorm/hypnovel on the TMR were not significant for [^99m^Tc]-HL91 whilst the impact of ketamine/xylazine use was less pronounced compared to [^18^F]-FMISO and [^64^Cu]-CuATSM. By and large, [^99m^Tc]-HL91 tumor uptake could only be altered significantly by administration of 100% oxygen. The latter was certainly not the case for [^18^F]-FMISO and [^64^Cu]-CuATSM. The TMR for both compounds was reduced significantly when either oxygen or anaesthetics (isoflurane in air, ketamine/xylazine or hypnorm/hypnovel) were introduced. Whilst for [^18^F]-FMISO no further decrease was measured when both isoflurane and oxygen were administered, [^64^Cu]-CuATSM did show an additional, subtle but significant, decrease in TMR. Moreover, when using the same anaesthetic regimes, the extent of TMR reduction was less pronounced for [^64^Cu]-CuATSM than for [^18^F]-FMISO (40–60% versus 70%, respectively). These differences in tracer behaviour might be an indication of their different hypoxia targeting mechanisms and/or hypoxia selectivity, a hypothesis that is also supported by the differences in TBR. Although the effects of anaesthesia are not as pronounced and universal for TBR as compared to TMR, they drop significantly for both [^64^Cu]-CuATSM and [^18^F]-FMISO when either oxygen or injectable anaesthetics are used and even more so when isoflurane was administered. Again, [^99m^Tc]-HL91 behaves differently. Both injectable anaesthetic regimes KXA and HHA raised the TBR as compared to awake mice breathing room air, whereas isoflurane did not seem to have any effect. Actually, only the use of oxygen reduced the TBRs. Despite the fact that the hypoxia targeting mechanism for [^99m^Tc]-HL91 is not yet known, these results might be another indication that passive diffusion is not the major pathway as was already suggested by Hsia et al [21].

To date, it is well established in small animal models, that radiation response can be improved and tumor hypoxia decreased by normobaric oxygen or carbogen breathing [22], [23]. Robinson SP et al reported that carbogen and 100% oxygen significantly increased arterial pO_2_, whilst only carbogen significantly increased arterial pCO_2_ and this without changing the arterial blood pressure. Additionally, they and others indicated that breathing high-oxygen content gases results in little additional O_2_ transport capacity as the haemoglobin is already almost completely saturated under air breathing conditions [24], [25]. Our results are congruent with the latter studies as only isoflurane in air but not the other anaesthetics caused an increase in arterial pO_2_, whilst pCO_2_ and sO_2_ remained constant for all tested regimes. This elevated arterial pO_2_ might relate to the reduced tumor hypoxia extent as measured by EF5 staining. Indeed, this could be backed up by others who either demonstrated a direct relationship between the extent of EF5 staining and tissue oxygenation status or confirmed an increase in tumor pO_2_ after 100% oxygen breathing [26], [27], [28]. Although corroborated by EF5 staining, OxyLite electrode measurements post imaging did not show the increased tumor oxygenation status when mice were breathing 100% oxygen. This could be attributed to the introduction of sampling errors as only a single measurement, and not pO_2_ histography, was performed due to the small size of murine tumors. Nonetheless, taken together all the above observations vindicate the lower TMR values for all three radiopharmaceuticals when oxygen was used as a carrier gas. Indeed, once [^18^F]-FMISO has entered the cell freely through passive diffusion, its specific retention in hypoxic tissues is dependent on the local oxygen tension [15]. Although the precise mechanistic details and the pO_2_ dependence for [^64^Cu]-CuATSM remains uncertain, the accepted mechanistic scheme as it stands today also involves bioreduction and trapping [29].

Although Lewis et al reported similar results to those presented here, i.e. that for animals breathing 100% oxygen, tumor uptake was 48% less than for those breathing air, others such as Matsumoto et al and Yuan et al found that [^64^Cu]-CuATSM tumor uptake was not decreased after carbogen breathing [30], [31], [32]. Fortuitously, these discrepancies are not necessarily due to a lack of hypoxia selectivity of the tested radiotracers but could be attributed to the use of carbogen [25], [33], [34].

Apart from the effects of the inspired gases, the anaesthetic drugs themselves also reduced the TMR values for [^18^F]-FMISO and [^64^Cu]-CuATSM. Moreover, these changes reflect a difference in uptake and not in contrast agent delivery as the dynamic blood curves showed no alterations for the tested anaesthesia regimes. Although the precise cause of these results was not the focus of this paper, they are not surprising. Indeed, among other parameters, variation in blood flow values alone may be responsible for altering the hypoxic cell fraction of experimental rodent tumors. Lee et al reported that Xylazine not only induces a strong hyperglycemic effect resulting in elevated plasma glucose levels but also causes a diuretic and natriuretic response [8]. Moreover, Menke et al showed that tumor blood flow in Ketamine/Xylazine sedated rats was reduced as compared to conscious animals [35]. While inhalational anaesthetics are much easier and safer to use, they do have the potential to influence hypoxia imaging results. Halogenated anaesthetics not only alter tumor metabolism and cardiovascular parameters, leading to hypercapnia, acidosis, reduced cardiac function, but they also reduce tumor pH and blood glucose levels [36], [37].

In conclusion, we focussed our study on oncology and more precisely on the preclinical development of radiotracers for non-invasive hypoxia imaging because of the heterogeneous and dynamic nature of hypoxia in tumors. However, the results could be applied to other disciplines and may be significant in the wider preclinical and clinical worlds. Our data showed that the use of anaesthesia can have profound effects on the experimental outcome. More importantly, all tested anaesthetics significantly reduced tumor-hypoxia uptake. Thus, the detectable differences between hypoxic and non-hypoxic tissue will be smaller under such anaesthesia leading to a reduction in statistical power and an increase in animal use to achieve good discrimination. Maximum tumor uptake was observed for conscious animals and as a result hypoxia imaging should employ conscious tracer uptake combined with anaesthesia only for the scan itself. This will not only benefit the experimental outcome but it will also better reflect the clinical situation as protocols could be more easily translated into the clinic. However, more often than not, anaesthesia cannot be avoided in preclinical studies but great care has to be taken in preclinical models of hypoxia as anaesthesia effects cannot be generalised across applications, let alone diseases. The presence of these anaesthesia related biological effects implies in practice that investigators should perform additional studies when the use of anaesthetics is required in order to characterize or rule out its confounding effects.
